# Coronary Artery Disease Empowerment Scale (CADES): Persian translation and psychometric properties

**DOI:** 10.1186/s12872-024-04369-x

**Published:** 2024-11-30

**Authors:** Amir Jalali, Amirhossein Naghibzadeh, Mojgan Rostami, Yasin Ahmadi, Mahbod Khodamorovati, Salam Vatandost, Niloufar Darvishi, Khalil Moradi

**Affiliations:** 1 Substance Abuse Prevention Research Center, Research Institute for Health, Kermanshah University of Medical Sciences, Kermanshah, Iran; 2 Student Research Committee, Kermanshah University of Medical Sciences, Kermanshah, Iran; 3 Department of Emergency and Critical Care Nursing, School of Nursing and Midwifery, Kermanshah University of Medical Sciences, Kermanshah, Iran; 4 Medical Department, The University of Georgia, Tbilisi, Georgia; 5 Clinical Care Research Center, Institute for Health Development, Kurdistan University of Medical Sciences, Sanandaj, Iran

**Keywords:** Coronary Artery Disease, Empowerment, Psychometric, Validity, Reliability

## Abstract

**Introduction:**

Coronary artery disease (CAD) is a leading cause of global mortality and a prevalent health issue in Iran. Assessing the empowerment of CAD patients during treatment and care is essential. However, no scale is available to measure empowerment in CAD patients in Iran. Therefore, this study aimed to evaluate the validity and reliability of the Persian version of the "Coronary Artery Disease Empowerment Scale" (CADES).

**Method:**

This methodological and cross-sectional study was conducted on patients with CAD from June 2022 to April 2023 in Kermanshah City, Iran. The scale was translated using the forward–backward translation method. Construct validity was examined using Exploratory Factor Analysis (EFA) with 190 samples and Confirmatory Factor Analysis (CFA) with 344 samples, both selected through convenience sampling. Internal consistency was assessed using Cronbach's alpha coefficient, and reliability was evaluated using the test–retest method. SPSS version 27 and LISREL version 8 software were utilized for data analysis.

**Results:**

The EFA and CFA results confirmed the instrument with three factors and 25 items. The model's main indicators in factor analysis were all above 0.9, indicating a good fit for the model. The Pearson correlation coefficient between the items and subscales with the main scale showed a direct and significant relationship. Additionally, Cronbach's alpha coefficient (0.813) and test–retest reliability (0.763) confirmed the reliability of the Persian version of the CADES.

**Discussion:**

The study's results suggest that the Persian version of CADES is both practical and acceptable for evaluating the empowerment of CAD patients. This tool can be relied upon as a valid and reliable method for assessing these patients' empowerment.

**Supplementary Information:**

The online version contains supplementary material available at 10.1186/s12872-024-04369-x.

## Introduction

Coronary artery disease (CAD) is the most common cardiovascular disease worldwide. It develops when plaque builds up inside the coronary arteries, causing them to narrow or become blocked. These essential blood vessels supply the heart with nourishment [1, 2]. Interruptions in the cardiac blood supply can trigger a heart attack or lead to serious conditions such as arrhythmias or heart failure [3]. Hence, CAD is characterized as a lethal disease.

The mortality and morbidity caused by this chronic disease are significant [4]. The prevalence of this disease in Iran is also high, accounting for 46% of total deaths and 20–23% of the disease burden [5]. Given the chronic nature of CAD, the main focus is on disease control and creating an acceptable quality of life for affected patients by empowering them to live with the disease [6]. Therefore, it is necessary to pay special attention to advancements in technology related to the control of CAD, as well as training healthcare professionals to provide services to these patients and empower them to effectively cope with various aspects of the disease, including psychological, physical, symptomatic, pharmacological, and other necessary measures that require active participation from the patient [7].

The concept of empowerment, which encompasses a set of necessary characteristics for patients to control their disease from various aspects such as intrapersonal, interactional, and behavioral, has been referenced in several texts. Numerous studies have explored the impact of enhancing this aspect of patients' characteristics in different chronic diseases [8, 9].

In exploring the available tools for empowering patients with CAD, we encountered an instrument devised by Lewin and Piper [10] that evaluates patients' comprehension of the care they receive, specifically within the context of empowerment. This instrument showed that while the majority of patients are content with the care provided, the notion of empowerment when dealing with acute illnesses does not hold considerable significance for them. However, this instrument failed to cover the diverse facets of the empowerment concept.

Accordingly, there has been a consistent recognition of the necessity for a dedicated instrument capable of extensively evaluating the multifaceted nature of the empowerment concept in patients with CAD. Considering the different capabilities required for patients to manage various chronic diseases, it is essential to have tailored and condition-specific assessments. Improper measurement can lead to incorrect and insufficient information, and potential corrective actions may not be taken in the right direction [11, 12]. Therefore, patients with CAD should be empowered to learn how to take care of their health and manage symptoms that may recur after intervention. Hence, an assessment tool specific to CAD is needed [13].

Recently, in South Korea, an instrument called the Coronary Artery Disease Empowerment Scale (CADES) was designed by Kim et al. in 2021 to assess empowerment in patients with CAD, and its validity and reliability have been confirmed [14]. This disease-specific instrument, comprising 25 items within three domains—self-determination, emotional self-regulation, and personal competence of disease management perception—successfully assessed the concept of empowerment, capturing its intrapersonal, interactional, and behavioral facets among patients with CAD [14].

Obtaining accurate knowledge about the empowerment of patients with CAD requires the development of trustworthy and universally applicable evaluation tools. However, in the context of Iranian society, reliable and valid Persian-language tools for assessing the empowerment of CAD patients are lacking. By reviewing the scale items designed in South Korea, it appears that these items are suitable for assessing CAD patients. Given the cultural and social differences between South Korea and Iran, as well as significant differences in healthcare systems, management, budget, and crisis response, a Korean tool developed for the Iranian society should undergo cultural validation and psychometric analysis. This study aimed to standardize the Persian version of CADES for the Iranian patient community and determine its psychometric characteristics.

## Method

The present cross-sectional and methodological study was conducted from June 20, 2022, to April 19, 2023, to assess the psychometric properties of the Persian version of the CADES among patients diagnosed with CAD. The study was carried out in two main stages: initially, the translation and cultural adaptation of the instrument, and subsequently, its psychometric evaluation.

### Participants and Setting

The study population consisted of patients with CAD from treatment centers and clinics in Kermanshah City. To ensure proper separation of the sample during the structure verification stage [15, 16], 190 patients were selected for the Exploratory Factor Analysis (EFA) stage [15], and 378 patients were chosen for the Confirmatory Factor Analysis (CFA) stage based on eligibility criteria and availability [15, 16]. The inclusion criteria for the study were as follows: an expressed interest in participation, a minimum of primary school education, a history of cardiovascular disease for at least three months, and a completed questionnaire with no more than 5% omissions. Out of the 378 patients initially selected, 34 questionnaires were excluded due to incomplete information, resulting in an analysis conducted on the remaining 344 questionnaires.

### Translation and cultural adaptation phase

#### Coronary Artery Disease Empowerment Scale (CADES)

The questionnaire under study was the Coronary Artery Disease Empowerment Scale (CADES), developed in 2021 by Kim et al. in South Korea. This instrument comprises three factors: self-determination (12 items), emotional self-regulation (7 items), and personal competence in disease management perception (6 items), totaling 25 items [14]. A 5-point Likert scale was employed to rate each item, with response options ranging from 'Strongly Disagree' to 'Strongly Agree.' Higher scores indicate higher levels of empowerment. The internal consistency of the total items was 0.93, indicating high reliability.

Initially, correspondence was made with the tool developers, and permission was obtained to conduct the study using the tool. Wild and colleagues' ten-step method was employed to evaluate both the content and response process during the cultural validation steps of the tool [17].

1. Preparation: We determined the instrument and obtained permission from its developers.
2. Forward Translation: After obtaining permission from the tool developers, two independent translators simultaneously translated the instrument from English to Persian using the forward method.
3. Reconciliation: The research team compared and reviewed the translated versions, merging them into a unified one.
4. Back Translation: The Persian version was back-translated into English simultaneously and independently by two translators who did not participate in the initial translation stage.
5. Back Translation Review: After reviewing the two English versions, a final integrated version was created and sent to the tool developers for feedback.
6. Harmonization: After comparing the translated version with the original tool, all vocabulary problems and inconsistencies were resolved. Finally, the coordination between the translated version and the original instrument was confirmed.
7. Cognitive Debriefing: The final Persian version was provided to 15 patients with CAD, who were asked to express any ambiguous points or possible issues (face validity).
8. Review of Cognitive Debriefing Results and Finalization: The final version was revised and refined based on their feedback.
9. Proofreading: The tool was approved by an expert in Persian language and literature after final editing
10. Final Report: After documenting all stages, the final version underwent psychometric evaluations.

#### Psychometric evaluation phase

In this phase, the evaluation concentrated on assessing the face validity, content validity, construct validity, and reliability of the Persian version of the CADES.

#### Face validity assessment (Qualitative and Quantitative)

In the qualitative phase, 15 patients with CAD who were not part of the initial sample evaluated the instrument's items for understandability, clarity, and appropriate correlation [18]. In the quantitative phase, this group of patients was requested to assess the importance of each item using a 5-point Likert scale (1 = Not important at all to 5 = Very important). After calculating the impact score for each item, those with an impact score greater than 1.5 were retained for further analysis [19].

#### Content validity assessment

##### Qualitative content validity

At this stage, the questionnaire was distributed among 14 members of the academic staff, researchers, and relevant specialists to determine the qualitative content validity of the instrument. They evaluated the scale's items for syntactical accuracy, phraseology, clarity, and cultural relevance to Iran.

##### Quantitative content validity

The instrument's content validity was evaluated using the Content Validity Ratio (CVR) and the Content Validity Index (CVI). The same 14 experts were invited to appraise the necessity of the instrument's items using a three-point Likert scale labeled 'Essential,' 'Useful but not essential,' and 'Unessential' for the CVR calculation. Furthermore, their recommendations for item wording revisions were compiled and integrated into the final version [20]. The Lawshe method was employed to determine the CVR of the instrument based on their ratings. The minimum acceptable CVR value, given the panel of 14 experts, was established at 0.51 [21].

The Content Validity Index (CVI) is used to evaluate the relevance of the instrument's items at both the individual item level (I-CVI) and the overall scale level (S-CVI). The same 14 experts were asked to evaluate the relevance of the CADES items by rating them on a four-point Likert scale ranging from 1 to 4 (1 = not relevant, 2 = somewhat relevant, 3 = quite relevant, 4 = highly relevant). The I-CVI was determined by calculating the ratio of experts who assigned a relevance rating of 3 or 4 to the total number of experts. Items that received a CVI value above 0.79 were classified as appropriate. Items with CVI values between 0.70 and 0.79 required revisions, while those below 0.70 were deemed inadequate and subsequently eliminated [22]. Furthermore, the S-CVI was calculated by averaging the CVI values of all items. An S-CVI value of 0.9 or higher indicates that the scale under assessment possesses strong content validity [23].

### Construct validity assessment

A test exhibits construct validity when the scores obtained from its administration are correlated with the intended concepts or theoretical constructs [21]. Both exploratory and confirmatory factor analyses were utilized to assess the construct validity of the Persian version of the CADES.

Out of 534 participants, and considering the significance of segregating the samples during each stage of construct validation [15, 16], a subset of 190 was selected for Exploratory Factor Analysis (EFA), and the remaining 344 were allocated for Confirmatory Factor Analysis (CFA). Previous studies have recommended that the dataset should be at least five times the number of items for EFA [24], and generally, it is advised to have a sample size of more than 200 participants for the CFA stage [25, 26]. Therefore, the number of participants in this study was deemed sufficient.

In this study, EFA was conducted using Varimax rotation. It is considered that the rate of explanation for the total variance should be greater than 40%, and the eigenvalues should exceed 1 to ascertain the factor structure of the scale [27, 28]. Additionally, factor loadings should exceed 0.3 to achieve an optimum construct [29]. The Kaiser–Meyer–Olkin (KMO) and Bartlett's tests were employed to assess the adequacy of the sampling. KMO values should be above 0.7, and the significance level for Bartlett's test should be less than 0.05 (*p* < 0.05) [30].

CFA validates the efficacy of each item in measuring the various dimensions of the scale. The criteria for the assessment of the model fit indices include the chi-square to degrees of freedom ratio (χ^2^/df) being less than three and the Root Mean Square Error of Approximation (RMSEA) being less than 0.08 [31]. Additionally, the Goodness of Fit Index (GFI) should exceed 0.90, the Comparative Fit Index (CFI) should be above 0.90, the Tucker-Lewis Index (TLI) should surpass 0.90, the Incremental Fit Index (IFI) should be greater than 0.90, and the Adjusted Goodness of Fit Index (AGFI) should be more than 0.80 [32].

### Reliability

The reliability of a questionnaire is reflected in the stability and consistency of its results [33]. Cronbach's alpha coefficient was used to measure the questionnaire's internal consistency. Furthermore, the questionnaire items were categorized into two sequences, odd and even, and the correlation between these two sequences' results was analyzed to determine the split-half reliability of the translated version.

The test–retest method was utilized to ascertain the questionnaire's temporal stability. A group of 35 patients was chosen in advance for this purpose, and their responses were re-assessed after two weeks [34]. It is generally acknowledged as satisfactory if the indices of Cronbach's alpha coefficient, split-half reliability, and test–retest reliability each meet or exceed the threshold of 0.70 [35, 36].

### Data analysis

In the present study, SPSS software version 27 and LISREL software version 8 were employed for data analysis. Descriptive statistics summarized the demographic data. The Waltz & Bausell index was applied to verify the quantitative content validity of the scale [37]. Exploratory and confirmatory factor analyses were used to verify the construct validity [38]. A *p*-value of less than 0.05 was deemed acceptable for the significance level of statistical tests. The skewness and kurtosis of the data distribution were utilized to assess the normality of the data in this study. The skewness for all items ranged from −0.16 to 1.01, and the kurtosis value ranged from −1.08 to 1.6. These values fall within the interval (−2, 2), indicating that the data distribution is almost symmetrical, as shown in Supplementary Table 1. The Cronbach's alpha coefficient and test–retest reliability [39] were used to confirm the scale's reliability. The internal consistency of the instrument was assessed using the Pearson correlation coefficient.

### Data collection procedure

After visiting each hospital and coordinating with the relevant authorities to secure the necessary permissions, the researcher employed a convenience sampling approach to select participants who fulfilled the study's inclusion criteria. Once the study's aims were clarified and consent was obtained, the questionnaires were directly distributed among patients during various shifts, including morning, afternoon, and night. The patients independently completed the questionnaires in a calm setting to ensure self-reporting. A total of 592 questionnaires were distributed among patients, out of which 534 questionnaires were analyzed, and 58 questionnaires were excluded from the study due to incomplete information.

## Results

### Descriptive results

In this study's EFA phase, 190 patients with CAD participated, with an average age of 56.71 ± 15.13 years and an age range spanning from 19 to 91 years. Among the participants, 58.2% were male, 71.6% were post-myocardial infarction patients, 29.3% had completed high school education, and 53.3% reported being in good health (Table 1).

**Table 1:** Demographic characters of participants in study (*N*: 534)

Variables		**CFA (344)**	**EFA (190)**
		N (%)	N (%)
Gender	Male	131(58.2)	202(58.7)
	Female	94(41.8)	142(41.3)
Diagnosis	Stable Angina	20(8.9)	26(7.6)
	Unstable Angina	44(19.6)	68(19.8)
	MI	161(71.6)	250(72.7)
Graduate Level	Elementary level	56(24.9)	82(23.8)
	Secondary Level	10.3(45.8)	160(46.5)
	Higher Education	66(29.3)	102(29.7)
Healthy feeling	Very bad	20(8.9)	32(9.3)
	Bad	56(24.9)	92(26.7)
	Good	120(53.3)	178(51.7)
	Very good	29(12.9)	42(12.2)
Job	Employed	135(60)	212(61.6)
	Non- Employed	90(40)	132(38.4)

In the CFA phase, the study included 344 patients. The average age of the participants was 57.06 ± 15.06 years, with an age range of 19 to 91 years. Among them, 58.7% were male, 72.7% had experienced a myocardial infarction, 29.7% possessed a high school diploma, and 51.7% described their health status as good (Table 1).

### Face validity

In assessing the qualitative face validity of the Persian version of the instrument, items 6, 17, and 22 were identified as requiring revisions to eliminate any ambiguity. These revisions were subsequently made and incorporated into the questionnaire. During the quantitative face validity assessment, all items achieved an impact score greater than 1.5, which led to the retention of all items.

### Content validity

In the qualitative content analysis, seven experts recommended revising four specific items (Items 6, 9, 18, and 23) to enhance clarity and comprehension. Following the review, these items were re-evaluated and subsequently confirmed.

The quantitative content validity was assessed using the Content Validity Ratio (CVR) for the entire questionnaire, which was 0.81, falling within the acceptable range of 0.67 to 1. Additionally, the Content Validity Index (CVI), calculated using the Waltz and Bausell index, was 0.80, with scores ranging from 0.75 to 0.92 (Supplementary Table 1).

### Exploratory Factor Analysis (EFA) of construct validity

An EFA was conducted with a sample of 190 participants. The KMO index for sampling adequacy was 0.914, and Bartlett’s test of sphericity was significant with a value of 5075.891 (*p* < 0.0001). Given that the KMO value exceeded the recommended threshold of 0.7, conducting a factor analysis is deemed justifiable [40].

**Fig. 1:**
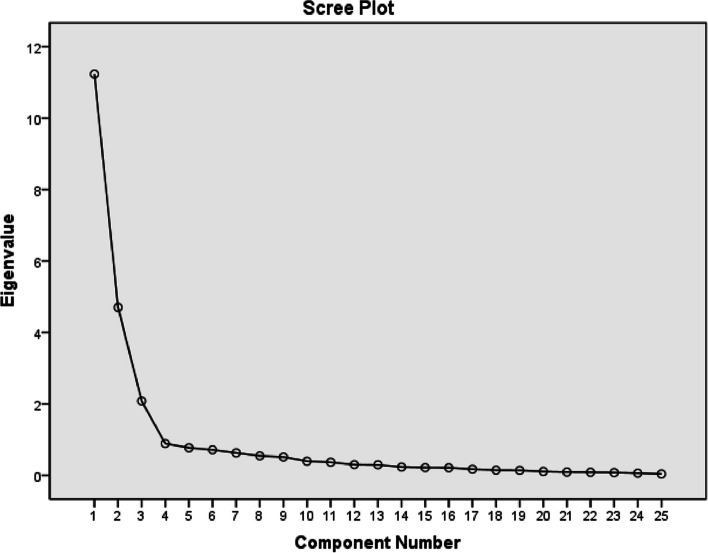
Cattell’s scree plot of the extracted elements of the scale

An EFA using principal component analysis (PCA) and Varimax orthogonal rotation identified a three-factor solution with eigenvalues greater than 1.0. This solution accounted for 72.66% of the total variance, surpassing the standard threshold of 40%. This result was also supported by the scree plot (Fig. 1).

As shown in Table 2 and Supplementary Table 2, all 25 items on the scale had factor loadings greater than 0.4 and were loaded onto three factors: 6 items in Factor 1, 12 items in Factor 2, and 7 items in Factor 3.

**Table 2:** Factor loadings of the CADES (*N*: 231)

Factor	Items		Factor		
			**1**	**2**	**3**
**Emotional self-regulation**	1	I can reduce my stress	-.293	**.810**	-.027
	2	I always do what is necessary to control my illness	.054	**.726**	-.220
	3	I use personal methods (prayer, mental relaxation, calm thinking, walking) to control my thoughts	-.339	**.607**	.236
	4	I will try to improve if I make a mistake in treating my illness	.067	**.939**	-.039
	5	I am trying to overcome my disease control problems	.046	**.482**	.179
	6	I can identify and address the causes of my stress	.086	**.864**	.158
	7	I can set up programs to control your illness	.359	**.575**	.044
	8	I create a balance between activity and rest to control my illness	.395	**.622**	.045
	9	In critical conditions such as sudden chest pain, I can take appropriate immediate action, such as taking medication	.471	**.657**	.021
	10	If needed, I can get financial support from a sponsor	.439	**.650**	.148
	11	I can definitely talk sincerely with my healthcare team about my emotions	.429	**.536**	.428
	12	I have a good relationship with my acquaintances (family, friends, etc.)	.431	**.695**	.334
Self-determination	13	I try to accept it when my condition worsens due to illness	.137	.026	**.896**
	14	I don't mind if those around me are aware of my illness	.164	.065	**.783**
	15	I am optimistic about my current situation	.336	.042	**.760**
	16	I'm trying to accept my illness	.277	.089	**.895**
	17	I am a person who can improve my health	.472	.231	**.668**
	18	I accept physical problems (such as weakness) resulting from illness	.350	.043	**.853**
	19	I have a goal in life that I want to achieve	.500	.108	**.686**
**Personal competence of disease management perception**	20	I am aware of the undesirable consequences (relapse, various heart diseases, etc.) that may occur in the future	**.762**	.132	.406
	21	I understand what to do when symptoms of an illness occur (such as taking medication, resting, etc.)	**.895**	.088	.273
	22	I am aware of what signs need to be treated again	**.850**	.114	.355
	23	I am well aware of my current medical condition	**.804**	.127	.446
	24	I know how to manage my illness (exercise, diet, quitting smoking, etc.)	**.858**	.168	.358
	25	I know how to treat my illness (medication, stenting, surgery, etc.)	**.868**	.077	.350
**Eigenvalue**			**11.233**	**4.701**	**2.082**
**Percentage of the variance %**			**25.430**	**23.686**	**22.950**

### Confirmatory Factor Analysis (CFA) of construct validity

**Fig. 2:**
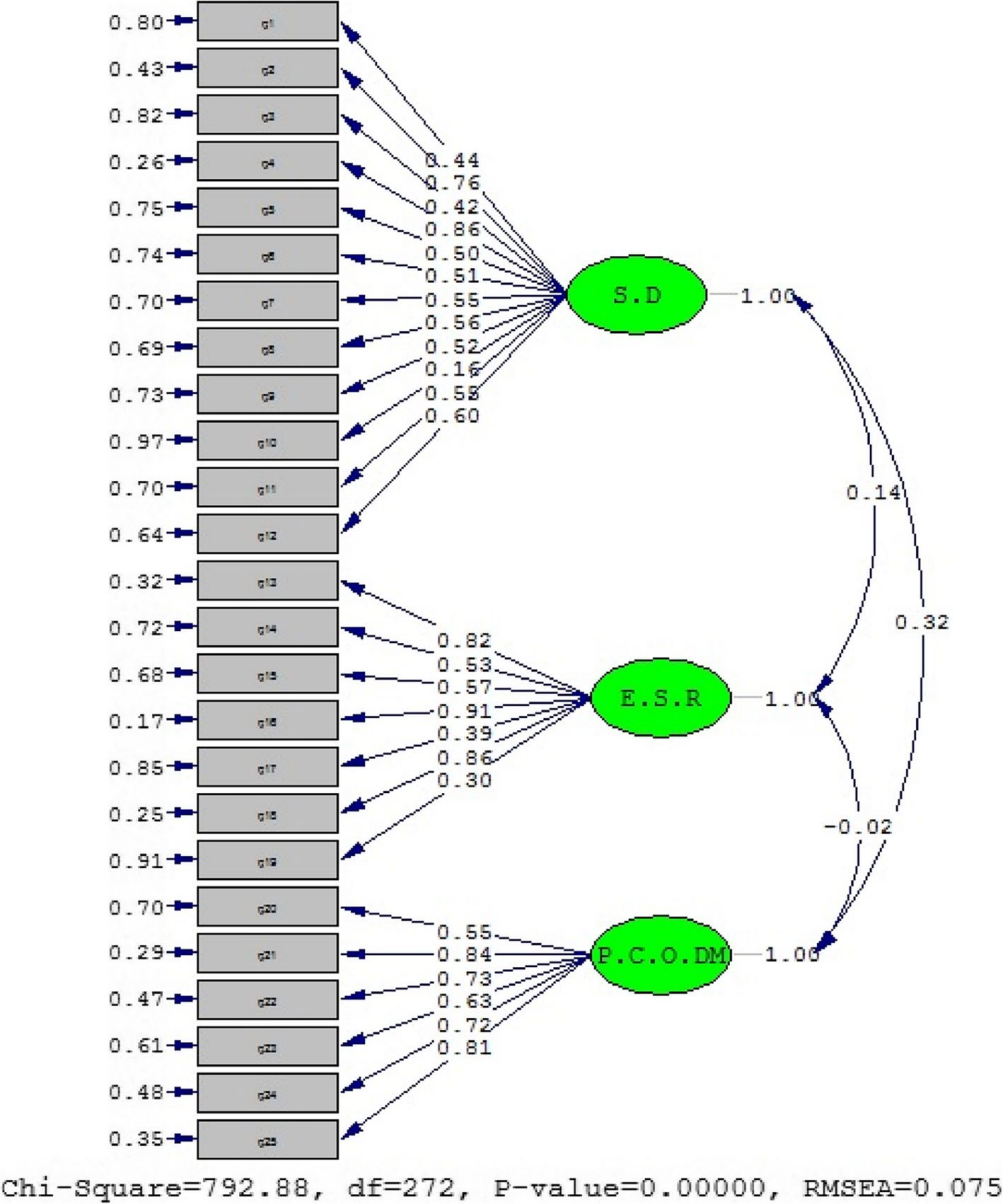
The three-factor CADE model was employed in the current study (with standardized coefficients)

A three-factor model was applied to a CFA of data from 344 patients, demonstrating a satisfactory fit. The fit indices were as follows: (*p* < 0.0001), RMSEA = 0.075, NNFI/TLI = 0.92, CFI = 0.90, GFI = 0.81, SRMR = 0.047, (DF = 272), (χ^2^/df = 2.91). The path diagram and the factor loadings from the CFA are displayed in Fig. 2. Furthermore, as detailed in Table 3, a significant and positive correlation between the subscales and the overall scale was indicated by Pearson's correlation coefficient.

**Table 3:** Pearson correlations for CADE domain scores

Factor	1	2	3
1. Personal competence of disease management perception	1		
2. Self-determination	.316^**^	1	
3. Emotional self-regulation	.269^**^	.206^**^	1

Correlations are latent factor correlation estimates from the CFA model. All correlations were statistically significant at **v_alue_ < .001

### Reliability analysis

The Cronbach's alpha value of the Persian version of the CADES was 0.813. The Cronbach's alpha values for the three dimensions of the questionnaire ranged from 0.739 to 0.809.

The split-half reliability of the translated questionnaire was 0.797. After two weeks, the questionnaire was administered again to a group of 35 patients. This follow-up assessment yielded a test–retest reliability coefficient of 0.763 (Table 4).

**Table 4:** Reliability assessment of the Persian version of the CADE

Total/sub dimension	Corrected item-total Correlation coefficient	Cronbach's alpha	Split-half reliability	Test–retest reliability
**Total**	.**433-.925**	**.813**	**0.797**	**0.763**
Self-determination	841-.925	.8		
Emotional self-regulation	.433-.866	.739		
Personal competence of disease management perception	.702- .917	.809		

## Discussion

The present study aimed to assess the psychometric properties of the Persian version of the Coronary Artery Disease Empowerment Scale (CADES) in Iran. The study results revealed that the Persian version of the instrument has suitable validity and reliability for assessing the empowerment of patients with CAD.

In this study, cultural validation was initially performed following the steps of Wild et al. [17]. The Persian version of the tool's face and content validity were also confirmed. In numerous studies dealing with cultural standardization and psychometric instruments, both face and content validity are frequently examined [14, 19, 22, 37]. In the Kim et al. study, which tested the Korean version of the tool, content validity was assessed using Waltz & Bausell indices [14]. In our study, we employed the CVR and CVI indices based on expert opinions for content validity.

To verify the construct's validity, the KMO test and its indicators demonstrated that EFA could be performed on the data. In numerous studies, EFA is employed to verify construct validity [14, 41]. In the current study, the KMO value was 0.914. Using the Varimax rotation method in factor analysis, we identified three factors that accounted for 72.66% of the total variance. The findings are similar to and consistent with those of the Korean version of the instrument [14] and the Korean version of the health empowerment scale for older adults [42]. This consistency might be due to cultural similarities between Iranian and Korean societies, despite their numerous differences. Generally, within Asian cultures, there is a prevailing tendency among patients to decline external help, opting for self-reliance.

The results of the EFA of the Persian version of the CADES showed that, in total, three factors with eigenvalues higher than one could explain 72.66% of the variance of 25 items. In the Korean version of the tool, three factors were able to explain 55.4% of the variance of 25 items [14], and in the Chinese version of the tool, four factors with eigenvalues higher than 1 were able to explain 62.38% of the variance of 25 items [43]. As can be seen, most of the studies obtained 25 items in exploratory factor analysis, but the number of instrument factors was somewhat different. In this context, it can be said that the culture of the studied society, along with the number of patients and their physical and mental conditions, has influenced their responses to the items.

The CFA results indicated that the Persian version of the tool, comprising three factors and 25 items, exhibited good fit indices. All values exceeded the critical threshold of 1.96, so no items needed removal. In the Chinese version, the tool model, consisting of 25 items and four factors, also demonstrated good fit indices [43]. The initial version of the instrument, developed and psychometrically tested in Korea, relied solely on EFA to confirm construct validity [14]. Both Chinese and Iranian societies appear to utilize the questionnaire effectively, according to the results of studies in this area. In this research, we employed both confirmatory and exploratory factor analyses due to the influence of contextual and cultural variables on results and questionnaire items.

The results of the present study showed that the correlation between the items and factors of the Persian version of the CADES in the studied population has a direct and significant correlation (*P* < 0.001). Additionally, the results indicated that the Persian version of the tool has appropriate and significant internal consistency. Cronbach's alpha coefficient (0.813) and test–retest reliability (0.763) confirmed the reliability of the Persian version of the CADES in the studied population. The results of the Chinese version of the tool also indicated good internal consistency, with a Cronbach's alpha coefficient of 0.928, Guttman's split-half coefficient of 0.777, and McDonald's omega reliability coefficient of 0.926 [43]. In the Korean version, the Cronbach's alpha coefficient was 0.93, indicating the appropriate reliability of the tool [14]. Reliability describes the consistency of results when measuring the same topic or indicator across multiple items. A strong internal correlation or uniformity between items indicates that they are consistently measuring the same construct, thereby increasing the scale's reliability [44].

Jafari Sejzi and colleagues evaluated the validity and reliability of the self-efficacy questionnaire for cardiovascular management, reporting an alpha coefficient of 0.80 [45]. Enhancing self-efficacy following the onset of cardiovascular disease is crucial for developing patients' skills to modify health behaviors, potentially reducing severe complications, hospitalizations, and surgeries [46]. Kim et al.'s study [14] identified a weak correlation between empowerment and self-efficacy in Korean patients with CAD, suggesting that self-efficacy functions as an empowerment approach in similar contexts. The concept of self-efficacy is closely tied to empowerment; it is essential to recognize that self-efficacy is both a facet of empowerment and an outcome, demonstrating its multifaceted nature [47]. A comprehensive literature review confirms that empowerment is a complex construct with multiple dimensions. In nursing, empowerment encompasses characteristics of both the patient and the nurse [48, 49]. This concept includes individual healthcare responsibilities as well as broader organizational and social responsibilities that enable individuals to take charge of their health [47]. The results can be discussed by considering the studied society, culture, participants' attitudes, and their relationships with others and society. Additionally, these factors may influence self-efficacy.

The primary component, 'Self-determination,' explained the most significant proportion of the instrument's total variance, accounting for 25.43%. This component incorporates behavioral dimensions, addressing strategies for stress management, spiritual competencies, and autonomous regulation. It also covers interactional dimensions, examining the roles of support networks (including relatives and acquaintances), financial assistance investigation, and dynamic engagement with medical practitioners. Research on empowerment has mainly focused on intrapersonal factors [50, 51]. This study provides a valuable addition by introducing a tool that integrates multiple elements. In the Korean version, the first factor accounted for approximately 25% of the total variance, encompassing 12 items [14]. In the Chinese version, the first factor is identified as self-determination, comprising nine items, while other items are categorized into different factors, including one labeled Seeking Support [43]. The explanation of the results suggests that various factors, such as the number of participants, the characteristics of the studied community, their perspectives, and the response method, may have influenced the findings.

The 'Emotional self-regulation' component, accounting for 23.686% of the total variance, is a crucial factor of the current instrument. This dimension contributed significantly to explaining the total variance, following the 'Self-determination' dimension. In the Korean version, this factor comprises seven items and explains 16.29% of the variance [14]. The Chinese version also uses the same seven items, with consistent results across all three studies [43]. This dimension includes items related to satisfaction with bodily alterations, autonomy in managing personal health, and self-initiated behavior [14, 43]. Emotional regulation refers to the spectrum of experiences, their processing, and the management of affective responses, which help manage emotional stressors in patients with chronic diseases [52]. This study emphasizes emotional and psychological dimensions as critical components [10]. Unlike the previously established tool for patients with CAD, which focused solely on interaction, this research offers a more comprehensive approach. The Persian version of the CADES is capable of evaluating empowerment, incorporating its intrapersonal, interactional, and behavioral dimensions in patients with CAD.

The third factor, 'Personal competence in disease management perception,' accounts for 22.95% of the total variance. In both the Chinese [43] and Korean [14] versions, this factor consists of six items and retains the same name. Studies support these findings, aligning with the current study's results. Patients with CAD tend to manage their health better when they understand their symptoms [53]. A study involving 270 Malaysian patients found that understanding illness, professional guidance, and access to health equipment are crucial for self-management [54]. Mosleh and Almalik's study demonstrated that understanding improves diet and exercise in CAD patients [55]. Therefore, understanding and self-management are essential for CAD patients. Tools designed for patients with chronic conditions (such as diabetes, COPD, and rheumatic diseases) have proven effective in aiding self-management [56, 57]. The 'Personal competence in disease management perception' component measures individual empowerment in understanding disease management, aiding patients in executing self-management roles, and maintaining self-regulation over time.

### Limitation

This investigation presents specific limitations that warrant consideration. Firstly, the study was confined to a cross-sectional analysis in a single province of Iran. The generalizability of the Persian version of the CADES to hospitals beyond this locale remains undetermined, necessitating further validation. Future research should extend to various provinces and cities and examine other validity methods, including predictive validity. Additionally, a limitation of this study was the variation in patients' motivation and the extent of their expressed opinions on different dimensions of empowerment, considering the diversity in their psychological characteristics and interpersonal behaviors.

## Conclusion

Empowerment, through the stimulation of self-awareness, provision of knowledge, and encouragement of patients, leads to increased motivation to participate in the management and control of health-influencing factors. A literature review indicated that research on the empowerment of patients with coronary artery disease (CAD) is limited in Iranian society. Therefore, it is necessary to have a valid and reliable instrument to evaluate the ability of CAD patients to manage their disease and self-care. This study validated the appropriateness of such an instrument for the CAD patient population, which can be beneficial for clinical, administrative, and research objectives.

## Supplementary Information

Supplementary Material 1.

## Data Availability

The datasets utilized in this research are accessible from the corresponding author upon a reasonable request.

## Abbreviations

CADES: Coronary Artery Disease Empowerment Scale
CVI: Content Validity Index
CVR: Content Validity Ratio
KMO: Kaiser–Meyer–Olkin
EFA: Exploratory Factor Analysis
CFA: Confirmatory Factor Analysis
TLI: Tucker-Lewis Index
NFI: Normed Fit Index
GFI: Goodness of Fit Index
RMSEA: Root Mean Square Error of Approximation
PC: Principal Components
SRMR: Standardized Root Mean Square Residual
KUMS: Kermanshah University of Medical Sciences
