# Supplementary material for: Coronary Artery Disease Empowerment Scale (CADES): Persian translation and psychometric properties
Source: BMC Cardiovasc Disord. 2024 Nov 30;24:693. doi: 10.1186/s12872-024-04369-x (PMC11608481; doi:10.1186/s12872-024-04369-x)
Supplement: Supplementary file 1 — Supplementary Material 1. [file 12872_2024_4369_MOESM1_ESM.doc]

**Supplementary table 1**. The ratio and index of content validity and Skewness and Kurtosis of the tool items

| **Kurt^d^** | **Skew^c^** | | **CVI^b^** | **CVR^a^** | **Items** | **No** |
| --- | --- | --- | --- | --- | --- | --- |
| **-1** | **-.33** | | **.83** | **1** | I can reduce my stress. | 1 |
| **.35** | **-.49** | | **.83** | **.83** | I always do what is necessary to control my illness. | 2 |
| **.88** | **-.63** | | **.83** | **.83** | I use personal methods (prayer, mental relaxation, calm thinking, walking) to control my thoughts. | 3 |
| **.18** | **-.34** | | **.83** | **.67** | I will try to improve if I make a mistake in treating my illness. | 4 |
| **.66** | **-.69** | | **.83** | **.83** | I am trying to overcome my disease control problems. | 5 |
| **-.95** | **-.18** | | **.75** | **.83** | I can identify and address the causes of my stress. | 6 |
| **.36** | **-.6** | | **.92** | **1** | I can set up programs to control your illness. | 7 |
| **.25** | **-.75** | | **.75** | **.83** | I create a balance between activity and rest to control my illness. | 8 |
| **.19** | **.69** | | **.67** | **.67** | In critical conditions such as sudden chest pain, I can take appropriate immediate action, such as taking medication. | 9 |
| **1.2** | **1.01** | | **.67** | **.83** | If needed, I can get financial support from a sponsor. | 10 |
| **.52** | **-.42** | | **.75** | **.67** | I can definitely talk sincerely with my healthcare team about my emotions. | 11 |
| **1.6** | **-.81** | | **.75** | **.83** | I have a good relationship with my acquaintances (family, friends, etc.). | 12 |
| **-.055** | **-.68** | | **.75** | **.67** | I try to accept it when my condition worsens due to illness. | 13 |
| **-1.08** | **-.16** | | **.92** | **.67** | I don't mind if those around me are aware of my illness. | 14 |
| **-.13** | **-.35** | | **.75** | **1** | I am optimistic about my current situation. | 15 |
| **.12** | **-.72** | | **.83** | **.83** | I'm trying to accept my illness. | 16 |
| **.31** | **-.69** | | **.92** | **.83** | I am a person who can improve my health. | 17 |
| **-.07** | **-.67** | | **.83** | **.67** | I accept physical problems (such as weakness) resulting from illness. | 18 |
| **-.58** | **-.28** | | **.75** | **.67** | I have a goal in life that I want to achieve. | 19 |
| **-.49** | **-.16** | | **.67** | **1** | I am aware of the undesirable consequences (relapse, various heart diseases, etc.) that may occur in the future. | 20 |
| **.57** | **.8** | | **.83** | **.67** | I understand what to do when symptoms of an illness occur (such as taking medication, resting, etc.). | 21 |
| **-.24** | **.029** | | **.83** | **.83** | I am aware of what signs need to be treated again. | 22 |
| **.024** | **-.26** | | **.83** | **.83** | I am well aware of my current medical condition. | 23 |
| **-.31** | **-.036** | | **.83** | **1** | I know how to manage my illness (exercise, diet, quitting smoking, etc.). | 24 |
| **.47** | **.86** | | **.83** | **.67** | I know how to treat my illness (medication, stenting, surgery, etc.) | 25 |
| **108.63** | 641.28 | **Multivariate** | | | |  |

a- Content Validity Ratio, b- Content Validity Index, c-Skewness is a measure of symmetry, or more precisely, the lack of symmetry, d-Kurtosis is a measure of whether the data are heavy-tailed or light-tailed relative to a normal distribution,

**Supplementary table 2:** Matrix of factor loadings of questionnaire questions on components after rotation

| **Items** | Component | | |
| --- | --- | --- | --- |
|  | 1 | 2 | 3 |
| q1 | -.293 | .810 | -.027 |
| q2 | .054 | .726 | -.220 |
| q3 | -.339 | .607 | .236 |
| q4 | .067 | .939 | -.039 |
| q5 | .046 | .482 | .179 |
| q6 | .086 | .864 | .158 |
| q7 | .359 | .575 | .044 |
| q8 | .395 | .622 | .045 |
| q9 | .471 | .657 | .021 |
| q10 | .439 | .650 | .148 |
| q11 | .429 | .536 | .428 |
| q12 | .431 | .695 | .334 |
| q13 | .137 | .026 | .896 |
| q14 | .164 | .065 | .783 |
| q15 | .336 | .042 | .760 |
| q16 | .277 | .089 | .895 |
| q17 | .472 | .231 | .668 |
| q18 | .350 | .043 | .853 |
| q19 | .500 | .108 | .686 |
| q20 | .762 | .132 | .406 |
| q21 | .895 | .088 | .273 |
| q22 | .850 | .114 | .355 |
| q23 | .804 | .127 | .446 |
| q24 | .858 | .168 | .358 |
| q25 | .868 | .077 | .350 |
